# Intraventricular injections of mesenchymal stem cells activate endogenous functional remyelination in a chronic demyelinating murine model

**DOI:** 10.1038/cddis.2016.130

**Published:** 2016-05-12

**Authors:** P Cruz-Martinez, S González-Granero, M M Molina-Navarro, J Pacheco-Torres, J M García-Verdugo, E Geijo-Barrientos, J Jones, S Martinez

**Affiliations:** 1 Neuroscience Institute, University Miguel Hernández – Spanish Council for Scientific Research (UMH-CSIC), Alicante, Spain; 2 Laboratorio de Neurobiología Comparada, Instituto Cavanilles, Universidad de Valencia, CIBERNED, Valencia, Spain; 3 IMIB-Hospital Universitario Virgen de la Arrixaca, Universidad de Murcia, Murcia, Spain

## Abstract

Current treatments for demyelinating diseases are generally only capable of ameliorating the symptoms, with little to no effect in decreasing myelin loss nor promoting functional recovery. Mesenchymal stem cells (MSCs) have been shown by many researchers to be a potential therapeutic tool in treating various neurodegenerative diseases, including demyelinating disorders. However, in the majority of the cases, the effect was only observed locally, in the area surrounding the graft. Thus, in order to achieve general remyelination in various brain structures simultaneously, bone marrow-derived MSCs were transplanted into the lateral ventricles (LVs) of the cuprizone murine model. In this manner, the cells may secrete soluble factors into the cerebrospinal fluid (CSF) and boost the endogenous oligodendrogenic potential of the subventricular zone (SVZ). As a result, oligodendrocyte progenitor cells (OPCs) were recruited within the corpus callosum (CC) over time, correlating with an increased myelin content. Electrophysiological studies, together with electron microscopy (EM) analysis, indicated that the newly formed myelin correctly enveloped the demyelinated axons and increased signal transduction through the CC. Moreover, increased neural stem progenitor cell (NSPC) proliferation was observed in the SVZ, possibly due to the tropic factors released by the MSCs. In conclusion, the findings of this study revealed that intraventricular injections of MSCs is a feasible method to elicit a paracrine effect in the oligodendrogenic niche of the SVZ, which is prone to respond to the factors secreted into the CSF and therefore promoting oligodendrogenesis and functional remyelination.

Demyelinating disorders, such as leukodystrophies and multiple sclerosis (MS), are neurodegenerative diseases characterized by the progressive loss of myelin that usually leads to a chronic demyelinated state, impairing normal axonal conduction velocity and ultimately causing neurological deficits.^1, 2^ This may be due to either an autoimmune attack (MS) or metabolic/genetic defects (leukodistrophy).^3, 4, 5^ The oligodendrocytes are crucial both for the metabolic support of the axons, ^6^ as well as the correct transmission of the nerve impulse, and therefore oligodendrocyte loss ultimately implicates neuronal degeneration.

New oligodendrocytes derive from the oligodendrocyte progenitor cells (OPCs), which are ubiquitously distributed throughout the brain parenchyma,^7, 8^ as well as from multipotent neural stem progenitor cells (NSPCs) present in the subventricular zone (SVZ). OPCs are capable of dividing throughout the lifespan and are activated when demyelinating damage is suffered.^9, 10, 11^ The OPCs are capable of differentiating into mature oligodendrocytes 7 days after an acute demyelinating lesion.^12^

MS can be divided into two phases: acute and chronic. During the acute phase, the nearby OPCs are capable of invading the lesion and remyelinate the damaged axons,^13, 14^ whereas in the chronic phase the cell's migratory and differentiating capabilities are affected, resulting in sustained and progressive demyelination.^15^ This impairment is partly due to the lack of factors that promote OPCs recruitment and stimulate remyelination, as well as the presence of inhibitory molecules.^16^ Previous studies in our lab,^17^ as well as others, have proven that OPCs may be activated and remyelination induced using bone marrow-derived mesenchymal stem cells (MSCs).^18, 19, 20, 21^ However, in the majority of the cases the effect was observed only locally in the immediate area surrounding the graft. In addition, most of the research has been performed using the experimental autoimmune encephalomyelitis (EAE) model, mainly focusing on the autoimmune inflammatory component rather than in the regeneration of the lost myelin. These studies have mixed results, possible due to the suboptimal application of MSC (reviewed in Kean *et al.*^22^). Therefore, an essential challenge for cell therapies in demyelinating diseases is to target not only the progression of the disease but also to achieve functional remyelination in several brain structures where myelin is lost.

In this context, the chronically demyelinated cuprizone model, where the blood–brain barrier (BBB) is intact,^23^ is an ideal alternative to study the remyelinating process without the participation of infiltrated peripheral immune cells. Thus, the aim of this study is to assess whether intraventricularly injected MSCs are capable of boosting the endogenous oligodendrogenic capacity, through a paracrine effect in the SVZ. The results of this work may give insight as to the mechanisms by which MSCs are capable of stimulating oligodendrogenesis in the NSPCs and ultimately induce functional remyelination in distant regions.

## Results

### Transplanted MSCs enhance the myelin content of the CC over time

**Figure 1:**
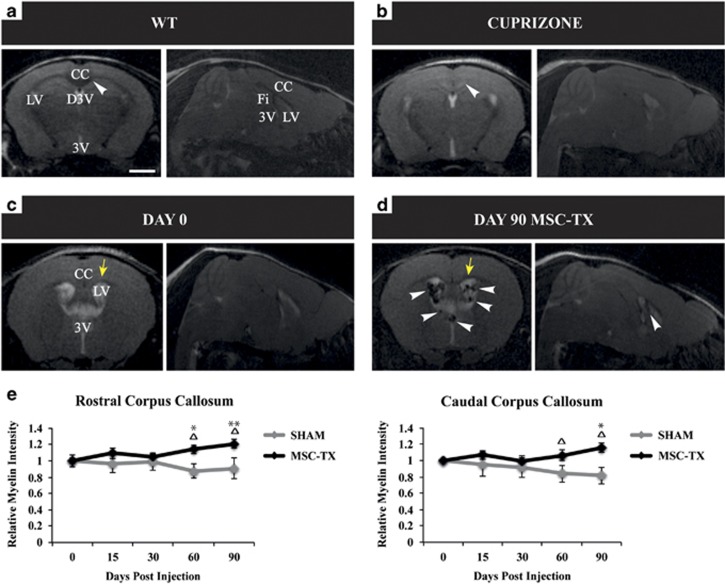
MRI and *in vivo* myelin content quantification. Representative brain T2-weighted images of WT (**a**) and chronic cuprizone-treated mice (**b**) in coronal and sagittal planes. Myelinated structures appear in black, gray matter in gray and CSF in white. The CC of the cuprizone-treated mice is depicted in a lighter gray color, corresponding with the demyelinated state (arrowheads in **a** and **b**). Representative images at day 0 before MSC transplantation (**c**) and 90 days after the injection (**d**). Ninety days after the injection, the MSCs were homogenously distributed and confined within the CSF of the ventricles. Arrowheads label the MSC appearing as black spots because of the contrast provided by the iron nanoparticles. Yellow arrows indicate the CC area where gray scale was normalized. (**e**) Quantification of the myelin density content. A significant increase in myelin was detected 2 months after transplantation in the MSC-treated group (statistically significant compared with SHAM (*P<0.05, **P<0.01) and compared with day 0 (Δ *P*<0.05)). *n*=6 in each treatment group. All data are reported as mean±S.E.M. D3V, dorsal third ventricle; Fi, fimbria; 3V, third ventricle. Scale bar, 1 mm (**a**–**d**)

In order to detect the level of demyelination, Klüver–Barrera staining, together with immunofluorescence (IF) for myelin basic protein (MBP) was performed. Although different patches of demyelination were observed in the fimbria/fornix and septum, the corpus callosum (CC) was rather homogenously affected among mice (see Supplementary Figure S1). As the CC is by far the largest fiber tract in the brain and is commonly affected in MS,^24, 25^ the study was mainly performed focusing on this structure. Approximately 300 000 MSCs in 2 *μ*l were stereotaxically injected into each LV of chronically demyelinated mice. Before transplantation, the MSCs were incubated for 24 h with iron nanoparticles, in order to allow their *in vivo* tracking by magnetic resonance imaging (MRI) analysis for up to 3 months. Also, myelin density can be visualized and quantified using this technique, as demyelination can be observed as dark patches within the CC (Figures 1a and b). The MSCs, which gave a negative contrast in the MRI images, were mainly detected in the LVs and in some cases in the third ventricle, but not in any other region of the CNS (Figures 1c and d). As for the myelin density, a significantly higher (*P*<0.05) level of myelin density content was observed in the cell-treated group starting at 2 months of age when compared with themselves at earlier time points as well as compared with the sham controls (Figure 1e).

### MSC intraventricular injections increase the number of OPCs and mature oligodendrocytes in the demyelinated CC

**Figure 2:**
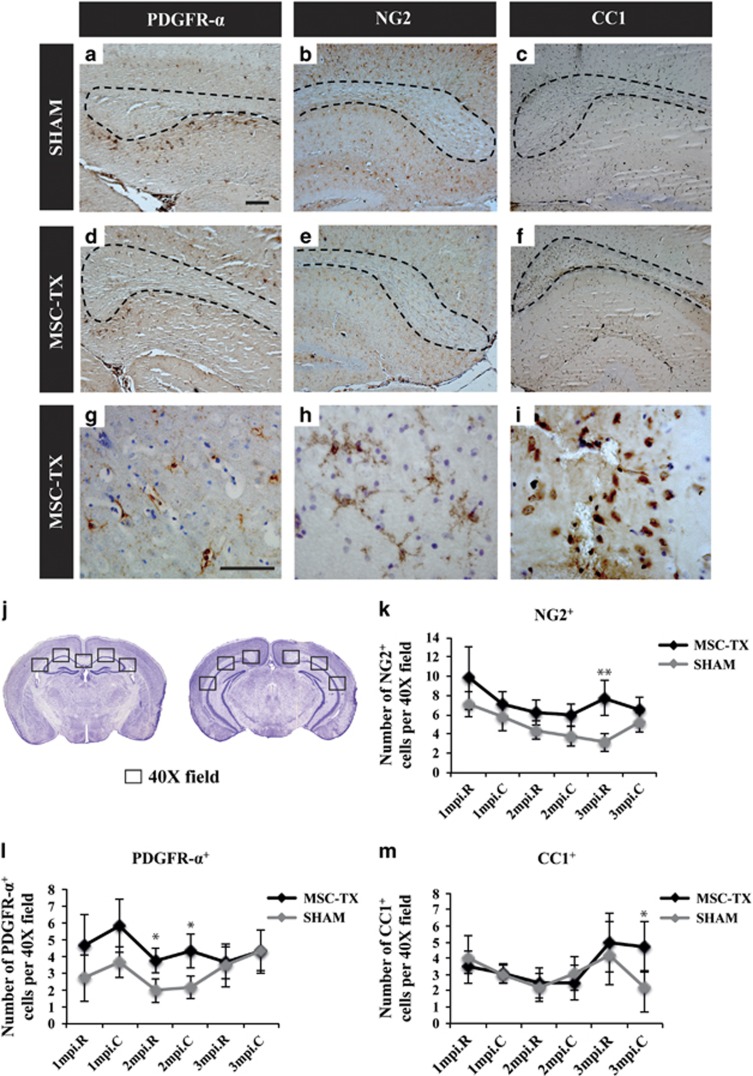
Quantification of OPCs and mature oligodendrocytes in the CC. (**a**–**f**) Immunostained brain coronal sections of the CC of stem cell-treated mice and SHAM for several OPC (PDGFR-*α*, NG2) and mature oligodendrocyte (CC1) markers, and counterstained with cresyl violet. The border of the CC is illustrated with a dotted line for better visibility. (**g**–**i**) Close-up images of the same coronal sections representing the typical morphology stained with each marker. (**j**) Schematic representation of mice brain sections containing the most rostral (midline-crossing) and caudal (non midline-crossing) CC. Quantification was performed in each 40X field within the CC of consecutive coronal brain slides as represented in the images. Adapted from Allen Brain Atlas (http://www.brain-map.org). Quantification of OPCs and mature oligodendrocytes at different months after MSC transplantation resulted in an increase of OPCs (NG2+ and PDGFR-*α*+) in the MSC-treated group 2 months after transplantation (**k** and **l**) and of mature oligodendrocytes (CC1+) in the most caudal CC (**m**). Statistically significant compared with SHAM (*P<0.05, **P<0.01). *n*=4 per group for each time point. All data are shown as mean±S.E.M. Scale bar, 100 *μ*m (**a**–**f**); 50 *μ*m (**g**–**i**)

The number of oligodendrocytes was calculated at different stages of differentiation within the rostral and caudal CC (Figure 2) over time (30–60–90 days after MSC transplantation). The average cell number per section was calculated by counting labeled cells in five random 40X fields per section in the rostral CC and three random 40X fields per section in each hemisphere of the caudal CC (Figure 2j). A significant increase was observed (*P*<0.05) in the number of OPCs (NG2^+^, PDFGR-*α*^+^) in the CC of the MSC-treated group compared with the SHAM, as early as 2 months after transplantation (Figures 2a, b, d, e, g, h, k and l) as well as a significant increase (*P*<0.05) in the number of mature oligodendrocytes (CC1^+^) 3 months after the injection at the caudal-most region of the CC (Figures 2c, f, i and m). This may be due to the paracrine effect of several trophic factors, which are likely to be stimulating the differentiation of NSPCs of the SVZ toward OPCs, their migration toward the CC and their final differentiation, whereas simultaneously activating pre-existing (parenchymal) OPCs in the nearby areas.

### Grafted MSCs remain primarily in the CSF, overexpressing trophic factors and with little penetration into the brain parenchyma

**Figure 3:**
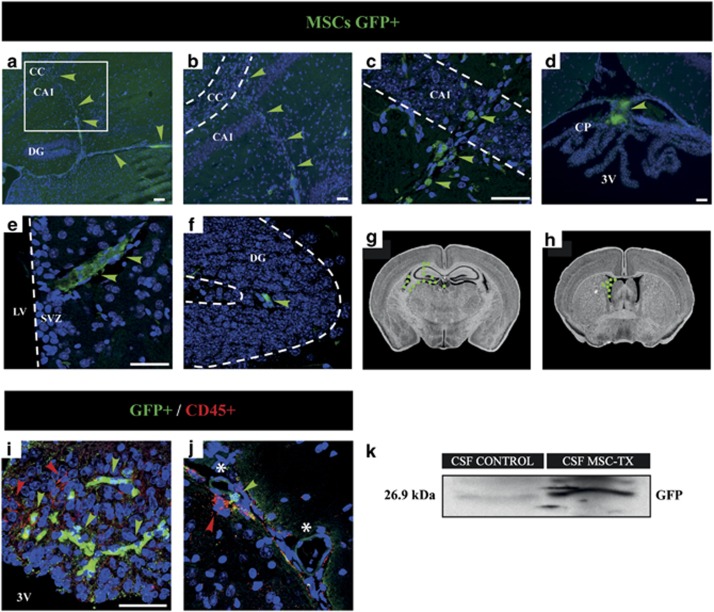
Location of intraventricularly transplanted MSCs and trophic content in the CSF. MSCs obtained from GFP transgenic mice were injected in the lateral ventricules of cuprizone-treated mice. IF analysis for GFP (green) were performed 3 months post-injection to detect the MSC location. The GFP+ MSCs were found penetrating into several brain structures such as the hippocampus (HP) (**a**–**c** and **f**), CC (**a** and **b**), SVZ (**e**) and attached to the choroid plexus (**d**). (**b**) Close-up region of a pathway from the HP to the CC (inset in **a**). Arrowheads indicate the location of MSC. The border of the CC, CA1, DG and SVZ are illustrated with a dotted line. (**g** and **h**) Schematic representation of coronal brain sections showing the location of the MSC after transplantation. (**i** and **j**) Double immunostaining for GFP (green) and CD45 (red). The MSC GFP+ appear surrounded by few macrophages (CD45 high) or microglia (CD45 low) as well as in close contact with blood vessels (**j**, asterisk). In all images, the blue staining corresponds to the nuclei (DAPI). Adapted from The Mouse Brain Library (http://www.mbl.org). (**k**) Western blot analysis of the CSF confirmed the presence of the MSC in the MSC-treated group 3 months post-injection. *n*=4 independent experiments and *n*=5 in each treatment group. CA1, cornus amonis 1; CP, choroid plexus; DG, dentate gyrus; 3V, third ventricle. Scale bar, 100 *μ*m (**a** and **e**); 50 *μ*m (**b-d, f, g, h, i** and **j**)

Three months after transplantation, MSCs were still present within the CSF of the ventricles, as observed in the MRI images. Further experiments were performed in order to analyze the precise location of these cells. To this end, both green florescent protein (GFP) transgenic mice, as well as wild-type C57BL/6 were used as donors to obtain the MSCs that were then injected into C57BL/6 cuprizone-treated mice. Immunohistochemistry (IHC) analysis (Figures 3a–j) were conducted 3 months post-injection and the CSF of both GFP+ MSC-treated and SHAM group was extracted at the same time point to analyze the presence of the GFP protein by western blot. The results indicated that the MSCs were still present within the CSF of the transplanted mice as shown by the expression of GFP protein (Figure 3k). Moreover, some cells were found attached to the choroid plexus (Figure 3d). In some animals, they were found penetrating into several brain structures sometimes forming long chains of migrating cells, such as in the hippocampus and CC (Figures 3a and c and f), and the lateral SVZ (Figure 3e). MSCs were detected in close contact with blood vessels (Figures 3c, e and j), possibly contributing and promoting angiogenesis, as it has been previously described.^26^ On rare occasion, MSCs (GFP+) were detected surrounded by macrophages or microglia (CD45+) (Figures 3i and j). In all cases, the regular morphology of the cell body, and the absence of cells with more than one nucleus indicated that there was no sign of trans-differentiation or cell fusion.

**Figure 4:**
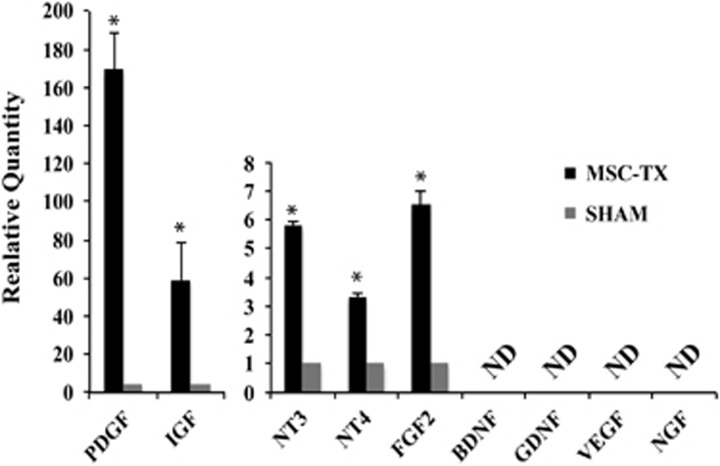
Quantitative PCR analysis of gene expression in the CSF. Overexpression of several trophic factor-encoding genes in the CSF of the MSC-treated group compared with SHAM. Statistically significant compared with SHAM (*P<0.0001). *n*=5 in each treatment group. All data are reported as mean±S.E.M.

Similarly, the trophic factor content of the CSF was analyzed 3 months after MSC transplantations. CSF was extracted from both MSC and SHAM-operated mice and subsequently analyzed by quantitative real-time PCR (qPCR) for different trophic factors. As a result, *PDGF, IGF, NT3, NT4* and *FGF2* genes were greatly overexpressed (*P*<0.0001) in the CSF of the MSC-treated group compared with the SHAM (Figure 4), which are the potential candidate factors that could, at least in part, be stimulating remyelination. Moreover, it seems reasonable to think that the MSCs previously detected in the CSF (Figures 1d and 3m) are responsible for this expression, whereas the low, almost nonexistent signal detected in the SHAM group could be due to the cellular debris that remained when the CSF was extracted.

### Axonal conduction velocity is increased in the CC of grafted mice over time

**Figure 5:**
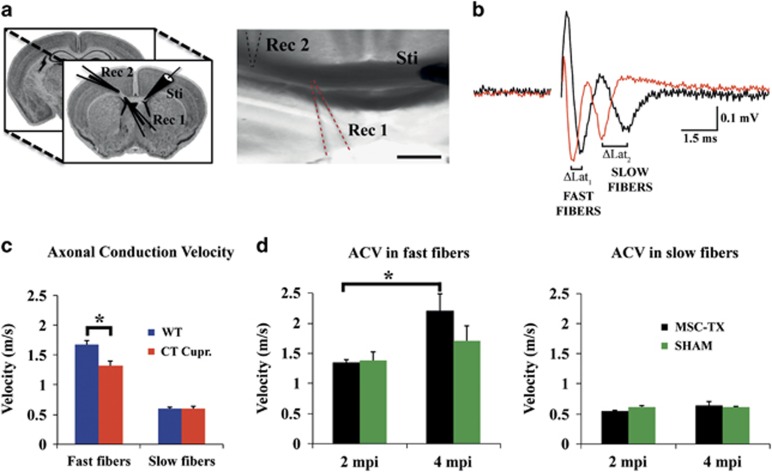
Intraventricular injections of MSC increase axonal conduction velocity over time. (**a**) Schematic representation of the brain slices corresponding approximately to plates 25–31 and 40–46 in the atlas of Franklin and Paxinos,^27^ and details of the position of the stimuli and two recording electrodes. (**b**) Typical CC CAP from recording electrode 1 (red), and recording electrode 2 (black); two fibers were detected: fast-conducting (thicker myelin sheaths) and slow-conducting fibers (thin and/or non-myelin sheaths); ΔLat (difference in latency between both peaks). (**c**) Histogram depicting the axon conducting velocities in the WT (blue) and cuprizone mice model (red). The cuprizone produced a significant decrease in the conduction velocity of the fast fibers when compared with the WT. (**d**) Histogram depicting the axonal conduction velocity over time of the MSC-treated (black) and SHAM groups (green). Conduction velocity of the slow fibers did not change in any experimental condition. Statistically significant compared between different time points (*P<0.05). *n*=4 in MSC-TX and CT cuprizone groups, *n*=8 SHAM and WT groups. All data are reported as mean±S.E.M. Scale bar, 300 *μ*m (**a**)

In order to characterize the functional consequences of the MSC treatment, compound axon potentials (CAPs) were recorded in callosal axons (Figure 5a). Coronal brain slices with midline-crossing segments of the CC, corresponding to specific Franklin and Paxinos^27^ atlas-based plates (see Materials and methods section) were used for recording, with a stimulus and two recording electrodes placed as shown in (Figure 5a). Two downward phases of the CAP corresponding to fast- and slow-conducting fibers were observed, probably representing fast depolarization from large, myelinated axons and slower depolarization from non-myelinated axons, respectively. Typical voltage recordings are shown in Figure 5b.

First, it was observed that the cuprizone produced a decrease in the conduction velocity of the fast fibers, whereas the slow fibers, corresponding to the non-myelinated axons presented normal values when compared with WT, as it was expected because of the fact that cuprizone only affects the oligodendrocytes and subsequently the myelinated axons (Figure 5c). Regarding the stem cell-treated mice and the SHAM group, the conduction velocity was recorded 2 and 4 months after MSCs transplantation. No significant differences were detected between the MSC treated and the SHAM animals at any time point for any fiber type, however, when the same group of animals were compared over time, the axonal conduction velocity was significantly increased in the MSC group (*P*<0.05). This difference was only observed in the fast conduction fibers, whereas the conduction velocity of the slow fibers did not change (Figure 5d). In conclusion, the electrophysiological analysis corroborate our previous data, indicating that the increased oligodendrocyte number and myelin content over time in the CC, correlated with functional recovery, observed by a significant improvement in the axonal conduction velocity of the MSC-treated group.

### MSC transplantations increase myelin thickness, decreasing the g-ratio of callosal axons

**Figure 6:**
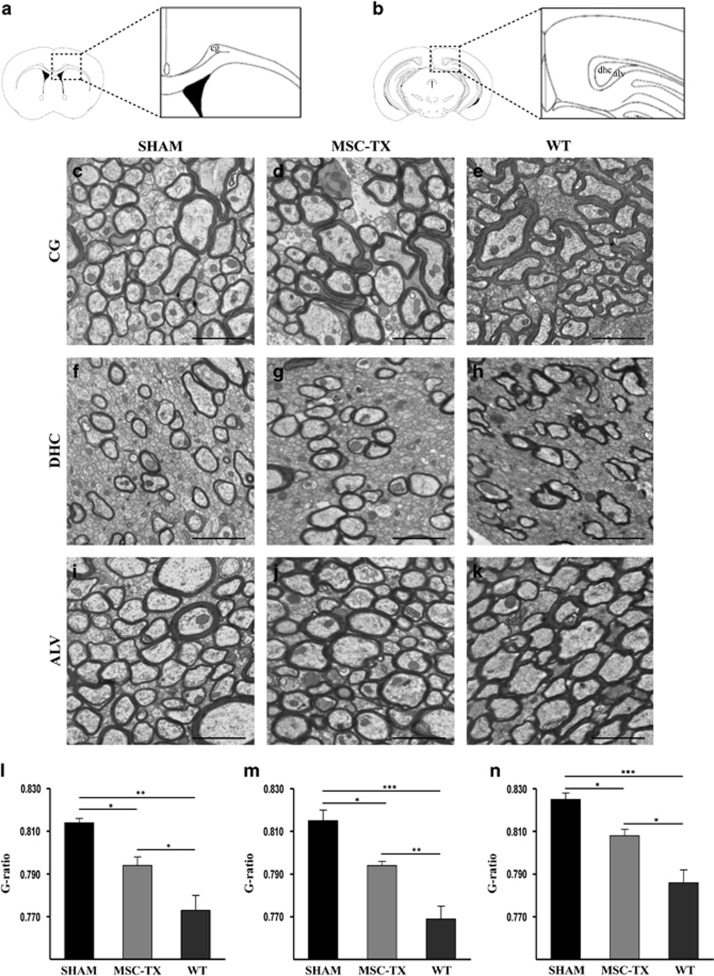
Quantification of myelin thickness by G-ratio analysis in three telencephalic areas. Coronal section scheme of anterior telencephalic level (**a**) and posterior telencephalic level (**b**) with a magnified image of these areas. Representative electron micrographs of cingulate fascicle (cg) (**c**–**e**), dorsal hippocampal commissure (dhc) (**f**–**h**) and alveus of the hippocampus (alv) (**i**–**k**) in Sham, MSC-TX and WT mice respectively. The graphs (**l**–**n**) depict the G-ratio quantification in cg, dhc and alv regions (*P<0.05, **P<0.001, ***P<0.001). *n*=4 in each treatment group. All data are reported as mean±S.E.M. Scale bar, 2 *μ*m (**c–k**)

Apart from the functionality of the newly formed myelin, it was of vital importance to further asses the integrity of its ultrastructure, calculating the axonal diameters, myelin thickness and mean g-ratio of myelinated and unmyelinated axons by electron microscopy (EM) analysis. To this end, the ratio between the inner axonal area and the outer myelin area (g-ratio), a gold standard for the assessment of demyelination and remyelination in experimental models, was therefore calculated. Three telencephalic areas, where abundant myelinated axons are present, were studied: *cingulate fascicle* (cg), in the anterior levels of the telencephalon (from Bregma 1.10 to −0.10 mm) (Figures 6a and c–e), *dorsal hippocampal commissure* (dhc) and *alveus of the hippocampus* (alv), two regions of the posterior levels (Figures 6b and f–k). The results revealed that the stem cell-treated group presented a significantly thicker myelin sheath than SHAM control mice in all the regions analyzed, and consequently lower g-ratio values. In addition, significant differences in the mean g-ratio when compared with the WT were observed in both groups, with a significantly higher difference in the case of the SHAM animals (Figures 6l and m).

### MSCs stimulate the proliferation of the NSPCs in the adult SVZ *in vivo* and activate proliferation and survival signaling routes in NSPCs *in vitro*

**Figure 7:**
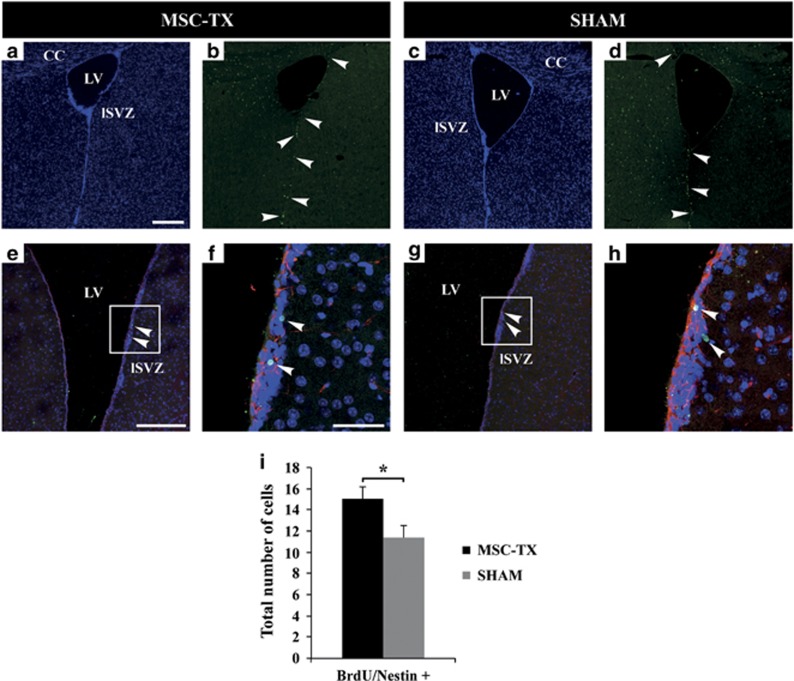
Increased proliferation of NSPCs in the adult SVZ. (**a–d**) Representative images of proliferating cells within the V-SVZ. Immunostaining for BrdU+ (green) and nuclei (DAPI, blue) is shown. Arrowheads indicate BrdU+ proliferating cells. (**e** and **g**) Representative images of proliferating BrdU/Nestin+ NSPCs in the SVZ. (**f** and **h**) Close-up regions were the cells were detected (inset in **e** and **g**, respectively). Arrowheads indicate double immunostained cells. (**i**) Histogram shows a significant increase in the total number of BrdU/Nestin+ cells per SVZ of MSC-treated mice compared with SHAM. Statistically significant compared with SHAM (*P<0.05). *n*=6 in each treatment group. All data are reported as mean±S.E.M. Abbreviations: lSVZ, lateral SVZ; Scale bar, 200 *μ*m (**a**–**d**); 200 *μ*m (**e** and **g**); 50μm (**f** and **h**)

Mammalian neurogenesis in the adult has been demonstrated to be mainly restricted to the SVZ of the lateral ventricles (LVs) and the subgranular zone (SGZ) of the dentate gyrus in the hippocampus.^28, 29^ Also, it has been reported that *in vitro* MSCs are capable of instructing an oligodendrogenic fate decision on adult NSPCs.^30^ Therefore, based on our previous findings, we hypothesized that the remyelination observed could be due to the enriched neurotrophic environment created by the MSCs, exerting a paracrine effect, activating endogenous neurogenesis and possibly an oligodendrogenic fate decision on the NSPCs of the SVZ, which are in direct contact with the cerebrospinal fluid (CSF) through their apical end and primary cilia. To corroborate this, 5-bromo-2-deoxyuridine (BrdU) incorporating assay was performed, immediately after MSC transplantation. Mice were given BrdU in the drinking water for 2 weeks just after transplantations, and killed 2 weeks later, to analyze the proliferation in the adult SVZ (Figures 7a–h). As a result, a significant increase in the total number of proliferating NSPCs (Nestin/Brdu+) was detected in the SVZ of MSC-treated mice when compared with SHAM (Figure 7i).

**Figure 8:**
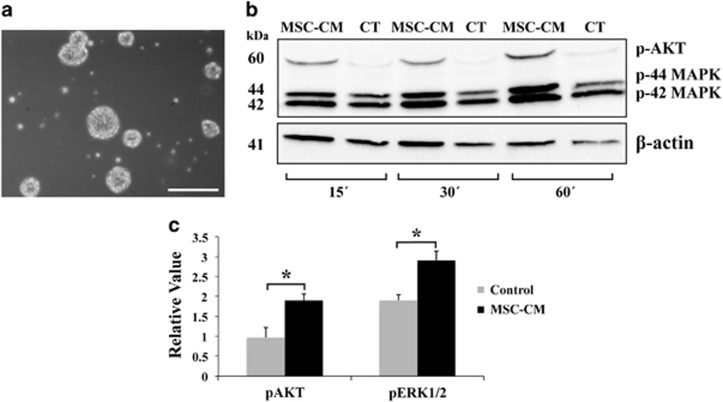
Western blot analysis of the activation of AKT and MAPK pathways induced by MSC-CM. (**a**) Undifferentiated NSPC grow in suspension, forming neurospheres just before treatment with MSC-CM. (**b**) Western blot images taken in the same gel for p-AKT and p-44/42 MAPK in non-conditioned (CT) and MSC-CM corresponding to different NSPC cultures at 15′, 30′and 60 min after exposure to the conditioned medium. (**c**) Histogram corresponding to the gel density analysis of CT and MSC-CM for p-AKT and p-44/42 MAPK after 15 min of exposure. Statistically significant compared with control (*P<0.05). *n*=4 independent experiments. All data are reported as mean±S.E.M. Scale bar, 500 *μ*m (**a**)

In order to further confirm these observations *in vivo*, the capacity of the MSCs to activate survival and proliferation routes *in vitro* was tested. To this end, NSPCs were cultured in the presence of MSC-CM and the activation of the phospatidylinositol-3 kinase (PI3K)/Akt and mitogen-activated protein kinase (MAPK)/extracellular signal-regulated kinases 1/2 (Erk1/2) signaling pathways was analyzed by western blot. These signal cascades have been shown to be involved in promoting neurogenesis, neuroprotection, oligodendrocyte fate decision and OPC differentiation, as well as inhibiting apoptosis.^31, 32, 33, 34, 35, 36, 37, 38, 39, 40, 41^ Thus, phospho-AKT and phospo-44/42 MAPK activation was studied at 15 min after adding MSC-CM to the NSPC cultures (Figure 8a), and compared with control cultures where MSC non-CM was added. As a result, both phosphorylated-AKT and phosphorylated-ERK1/2 protein expression levels changed significantly compared with controls (Figure 8c). This observation indicated that MSC-CM was capable of activating both routes in the NSPC *in vitro*, and therefore corroborate the initial hypothesis.

## Discussion

The adult CNS maintains a certain endogenous potential to repair myelin damage. This regenerative function is, however, insufficient in chronic MS, where progenitor cells either fail to be recruited into the lesion sites^42^ or remain in a quiescent state, uncapable of differentiating into mature oligodendrocytes.^43, 44, 45^ The microenvironment of chronic demyelinated lesions also has an important role, as it inhibits remyelination.^46^ This inability to repair the damaged myelin is a major obstacle for MS treatment, leading to axonal and neuronal damage.^1, 2^ Therefore, current MS treatments mostly focus on reducing the formation of inflammatory lesions within the CNS but do not enhance endogenous myelin repair. We believe that boosting the endogenous oligodendrogenesis, through appropriate cell therapies as presented in this work, may possibly lead to the development of significant repair strategies for the demyelinated CNS and complement the currently available immunosuppressive treatments.

In this regard, MSCs represent an attractive cell-based tool to treat demyelinating disorders. First, MSCs are fairly easy to obtain and expand, avoiding more invasive techniques. Second, MSCs can generally be used autologously. Third, MSCs exert stromal bystander immune-modulatory, neuroprotective and eventually remyelinating activities in the damaged CNS. Our group^17^ as well as others^18, 20, 47, 48, 49, 50, 51, 52, 53, 54, 55, 56, 57^ have demonstrated that *in vivo* transplantations of MSCs increase neuroprotection, modulate neuroinflammation, reduce demyelination and partially enhance functional recovery in MS models. However, the majority of the work has been focused in the EAE model after intraperitoneal or intravenous transplantation, with a certain degree of ambiguity in the efficacy of the transplants, possibly due to suboptimal application of MSC. In this context, intraventricular transplantation appears as an alternative method to administer the MSCs.

In this study, we used a variety of tools (immunohistochemical analysis, qPCR, western blot, MRI, electrophysiological recordings and EM) to study whether intraventricular transplantation of MSC in a chronic demyelinated model was a more feasible route of administration in terms of cell survival, engraftment, duration of the effect and mainly, the potential to stimulate the endogenous neurogenic/oligodendrogenic processes of the ventricular subventricular zone (V-SVZ). Our data revealed that intraventricular MSCs transplants were capable of stimulating remyelination (Figure 1), correlating with an increase in the number of OPCs and mature oligodendrocytes within the CC over time (Figure 2). However, it was crucial to confirm if the newly formed myelin was functional, as it is possible that the oligodendrocytes may not be capable of correctly placing the myelin layers around the damaged axons (reviewed Franklin^58^). Electrophysiological studies confirmed that, whereas no differences between the MSC-treated and SHAM animals in terms of conduction velocity were detected, there were significant differences between the groups when comparing themselves throughout time (Figure 5d). Moreover, this difference was only observed in the fast conduction fibers, whereas the conduction velocity of the slow fibers did not change, indicating that the myelination was directed only to the axons that were previously myelinated, instead of aberrantly myelinating the slow fibers (Figure 5d). Finally, myelination ultrastructure was analyzed by EM, revealing that the stem cell-treated group presented a significantly thicker myelin sheath than SHAM control mice in all the regions analyzed and consequently lower g-ratio values, similar to the values observed in the WT (Figure 6).

Interestingly, the results of the MRI (Figure 1d), IHC and western blot tests (Figure 3m) confirmed that the MSCs, when injected into the LVs, mainly remained in the CSF throughout the whole time of experimentation, with few cells integrating into the brain tissue (Figures 3a–j). On rare occasions, some of the MSCs detected in the brain parenchyma were observed near resident microglia and infiltrated macrophages (Figures 3i and j). Therefore, the only method that the MSCs may be acting upon the CNS is through the secretion of growth/trophic factors into the CSF, which would stimulate nearby progenitors, including those located in the V-SVZ. In this region, type B1-cells, which are NSPCs, bear a single primary cilium that projects into the ventricle.^59^ The function of this apical ending has been considered essential for growth factor, morphogen reception and transduction, and therefore for the proliferation, survival or differentiation of these quiescent cells.^60, 61, 62^ In fact, several studies have reported that the CSF flow has a major role in the proliferation^63^ and directional migration^64^ of these cells. Moreover, several groups reported the migratory potential of SVZ cells outside the rostral migratory stream and their capacity to generate mature oligodendrocytes in the adult brain.^9, 11^ Similarly, Aguirre and Gallo^65^ showed that the activation of SVZ progenitors, which can ultimately result in remyelination, depends on a variety of growth factors such as EGF.

These observations, together with our previous findings, lead us to hypothesize that the NSPCs of the V-SVZ may be responding to the soluble factors secreted by the MSCs (Figure 4) into the CSF, increasing their proliferative rate and inducing an oligodendrocytic fate decision. These newly generated OPCs would migrate to the lesioned areas and remyelinate the damaged axons. However, this increase in oligodendrocytes could derive not only from the SVZ, but possibly from the activation of pre-existing OPCs in the ventricular surface of the CC, which coincide with the dorsal pole of the LV. This hypothesis was confirmed by BrdU analysis, indicating an increase of proliferating NSPCs within the SVZ in the MSCs-treated mice (Figure 7). Finally, *in vitro* analysis showed an activation of PI3K/Akt and MAPK/Erk1/2 signaling pathways in the NSPCs because of the MSC-CM, routes that participate in processes of proliferation, differentiation, movement and survival; confirming that the MSCs were capable of activating and increasing NSPCs proliferation (Figure 8). Altogether, these results suggest that intraventricularly injected MSCs stimulate and protect the NSPCs of the SVZ, as well as possibly stimulating nearby pre-existing OPCs, activating the necessary mechanisms for functional remyelination.

In conclusion, these findings represent a major advancement for the management of chronic demyelinating neurodegenerative diseases. Ultimately, a deeper understanding of the underlying mechanisms in oligodendrocyte generation from NSPCs, as well as the individual effect of each trophic factor, may lead us to optimize the MSC-based therapy for the treatment of these pathologies.

## Materials and Methods

### Animal models

All experiments were performed in compliance with the Spanish and European Union laws on animal care in experimentation (Council Directive 86/609/EEC) and have been analyzed and approved by the Animal Experimentation Committee of our University. GFP (C57BL/6-Tg(ACTB-EGFP)1Osb/J) transgenic mice were used as donors, whereas C57BL/6 wild-type mice were used as hosts. Both animal strains were bred and maintained in our animal facilities.

### Cuprizone treatment to obtain the chronically demyelinated model

The approach used was similar to previously published reports.^17, 66^ Six-week-old C57BL/6 mice were given a diet with 0.2% cuprizone (w/w) (Sigma-Aldrich, St. Louis, MO, USA) for 12 weeks *ad libitum* in order to obtain a chronic version of toxicity-induced demyelination. Finely powdered cuprizone was mixed in the rodent chow and administered to the mice using specially designed feeders. After 12 weeks, the mice were used for different experiments and returned to normal chow until they were killed.

### Tissue culture

#### MSCs isolation and culture

The isolation and culture of MSCs was performed as in our previous studies.^17, 66, 67, 68, 69^ Briefly, the bone marrows of either transgenic GFP mice or C57BL/6 wild-type were extracted, disaggregated and cultured in plastic flasks. The following culture medium was used: Dulbecco's modified Eagle's medium (DMEM), (High Glucose) with GlutaMAX (BD Bioscience, San Diego, CA, USA), 15% fetal bovine serum (Biochrom, Berlin, Germany) and 1% penicillin/streptomycin (Gibco, Life Technologies, Baisley, UK). The stem cells were maintained for 3–4 weeks (passages 3–4), changing the media every 2–3 days, replating when needed, before proceeding to the surgical interventions.

#### NSPCs isolation and culture

NSPCs were isolated and cultured in a similar manner as published in Hutton and Pevny^70^ and Wachs *et al.*^71^ Briefly, NPSCs were isolated from newborn C57/BL6 mice. Newborn C57/BL6 mice mice were killed and brains extracted in a cell culture hood. The olfactory bulb, cerebellum and meninges were removed and the SVZ was microdissected. The tissue was then minced and treated with 1 mg/ml Collagenase XI (Sigma, St. Louis, MO, USA)/3 mg/ml Dispase (Gibco). After centrifuging to removing the supernatant, the resulting cellular suspension was cultured in Neurobasal medium (Life Technologies) supplemented with B27 (Gibco), 20 ng/ml EGF (Roche, Basel, Switzerland), 2 ng/ml bFGF (Roche), 0.7 U/ml Heparin (Sigma), 2 mM l-glutamine (Gibco) and 1% penicillin/streptomycin (Gibco) and plated in non-coated flasks. The culture was maintained for 10–15 days, changing half of the media every 2–3 days, replating when needed, before neurospheres were collected for experiments.

#### Preparation and use of MSC-conditioned medium (MSC-CM)

MSC-CM was collected 24 h after changing the culture medium of MSCs (passage 3) at 60–70% confluence. The MSC-CM was then filtered using a 0.22 *μ*m filter. Neurosphere cell suspension was collected, divided into two different conical tubes, centrifuged and the supernatant was discarded. The pellets were resuspended in either MSC-CM or the control medium (DMEM), (High Glucose with GlutaMAX), respectively. The cellular suspension was cultured in various Petri dishes and placed in an incubator. The culture was stopped at 15′, 30′ and 1 h by pipetting the cell suspension of each Petri dish into an eppendorf, and subsequently processed for protein analysis by western blot.

### Surgical procedures

#### MSCs transplantation and experimental groups

A total of 40 mice were injected with MSCs (*n*=12 for IHC, *n*=4 for IF, *n*=6 for MRI, *n*=12 for electrophysiology, *n*=4 for EM). For the sham-operated group (controls where only culture medium was injected), the same number of animals was used for each type of experiment. Before performing the surgery, the mice were injected intraperitoneally with a mix of anesthesia/analgesia containing 80–100 mg ketamine (Richter Pharma, Wels, Austria) and 10 mg xylazine (Laboratorios Calier, Barcelona, Spain) per kilogram body weight. The mice were placed in a stereotaxic apparatus (Stoelting, Wheat Lane Wood Dale, IN, USA), in order to perform the injections using the following coordinates: anteroposterior relative to bregma, mediolateral and dorsoventral from the surface of the brain: +0.5, 0.75 and 2.5 mm, respectively. The MSCs were injected using a 5 *μ*l Hamilton syringe (Reno, NV, USA). A volume of 2 μl, containing approximately 300 000 cells, was injected in a 4-min time lapse into each LV. After the injection, the incision was sutured and 30 ml of sterile PBS per kilogram body weight were injected subcutaneously to avoid dehydration. Mice were kept warm until they fully recovered. Then, buprenorphine (RB Pharmaceuticals, Slough, UK) was injected at a dose of 0.1 mg per kilogram body weight, and the animals monitored daily for at least 1 week. Finally, the mice were maintained until their use for experimentation (30–60–90 days post surgery for IHC, 0–15–30–60–90 days for MRI, 30 days for BrdU analysis, 60–120 days for EM and electrophysiology).

### Molecular biology techniques

#### Western blot analysis for NSPC protein extracts

Proteins were processed for western blot analysis to determine the relative levels of phospho-AKT (p-AKT) and phospho-ERK1/2 (p-ERK1/2). Neurospheres were lysed and the proteins separated by electrophoresis in 10% SDS-polyacrylamide minigels and transferred to nitrocellulose membranes (Protran, Whatman GmbH, Whatman Protran membrane, Sigma-Aldrich). Membranes were blocked and incubated with primary antibodies diluted in blocking solution overnight at 4ºC: rabbit anti-Akt phosphorylated (Ser 473, 1 : 1000, Cell Signalling), rabbit anti-ERK1/2 di-phosphorylated (Thr 44/42, 1 : 1000, Cell Signalling Technologies, Danvers, MA, USA) and mouse monoclonal anti-*β-*actin (1 : 15 000, Sigma) for control charge. After extensive washing, membranes were incubated with the HRP-conjugated goat anti-rabbit 1 : 5000 or HRP-conjugated horse anti-mouse 1 : 7000 (Vector Laboratories, Birmingham, CA, USA) diluted in 1% milk blocking solution for 90 min at room temperature. The membranes were developed with the Luminata Forte HRP substrate (Millipore, Millerica, MA, USA) and images taken with a LAS-1000 apparatus (Fuji, Tokyo, Japan). Quantifications and data analysis were performed using ImageJ (free software. http://imagej.net) as described by Hu and Beeton.^72^

#### Western blot analysis of CSF extract

Animals were anesthetized 3 months after MSC injection and placed in the stereotaxic device. The CSF was withdrawn from the LVs using a syringe, in the same coordinates where the MSCs were injected. A total of 20 *μ*l was taken from each animal (*n*=6). Age-matched sham-injected animals were used as controls. The samples were processed as previously commented in the western blot protocol, analyzing in this case the GFP protein content. Rabbit anti-GFP (1 : 5000, Molecular Probes, Eugene, OR, USA) was used as a primary antibody and HRP-conjugated goat anti-rabbit (1 : 7000, Vector Laboratories) as a secondary antibody. The same antibodies used for the control charge in the previous section were used.

#### qPCR of CSF extract

CSF of both MSC-transplanted and SHAM groups was analyzed for the gene expression of neurotrophic factors such as neurotrophin 3 and 4 (*NT3* and *NT4),* platelet-derived growth factor (*PDGF),* insulin growth factor *(IGF),* vascular endothelial growth factor *(VEGF),* brain-derived neurotrophic factor (*BDNF),* fibroblast growth factor 2 *(FGF2*), nerve growth factor (*NGF)* and glial-derived nerotrophic factor *(GDNF)*. Total mRNA of the cells was isolated using the Trizol protocol (Invitrogen, Carlsbad, CA, USA). A total of 5 *μ*g of mRNA was reverse-transcribed, and approximately 100 ng of cDNA was amplified by Real-Time PCR using Power SYBR Green Master mix (Applied Biosystems, Foster City, CA, USA). All the samples were run in triplicate using the StepOne Plus Real-Time PCR system (Applied Biosystems) and analyzed with the StepOne Software (Thermo Fisher Scientific). Analyses were carried out using the ΔC(T) method and calculated relative to GAPDH. The results were normalized with respect to the control condition, which presented a value of 1, using the same approach as in our previous report.^73^ The sequences of the primers used are shown in Supplementary Table 1. The resulting PCR products were placed in a QIAxcel apparatus (Qiagen, Hilden, Germany) and analyzed using the QIAxcel gel analysis software (QIAxcel Biocalculator, Qiagen).

### BrdU proliferation assay

MSC-transplanted and SHAM control mice were administered BrdU (Sigma) in the drinking water (1 mg/ml) daily for up to 2 weeks just after transplantation, followed by another 2 weeks with BrdU-free drinking water. Mice were then killed and brain sections obtained as previously described. Sections were double immunostained for BrdU and Nestin. Micrographs were taken with a Leica DM5500 laser scanning confocal microscope (Leica Microsystems, Wetzlar, Germany). Ten to 12 z-stack images (1-*μ*m Z-step) were taken and all the focal planes merged to visualize the maximum projection. Sixteen 14-*μ*m sections that were 84-*μ*m apart were selected, containing the SVZ. The total number of double immunostained cells (BrdU/Nestin+) per section and per SVZ was then calculated.

### IHC and stainings

The mice were anesthesized with isoflurane (Esteve Veterinary, Barcelona, Spain) and perfused intracardially with ice-cold 4% paraformaldehyde (PFA). The brain was carefully excised and kept in 4% PFA overnight. After fixation, the samples were dehydrated and paraffin-embedded overnight. Fourteen-μm coronal serial sections were obtained using a microtome (Microm HM335E, Walldorf, Germany), mounted on six parallel series and processed for stainings or IHC. Double-labeled IHC was performed using the following primary antibodies: anti-NG2 rabbit polyclonal IgG (1 : 150, Chemicon/Millipore, Billerica, MA, USA), anti-NG2 rabbit polyclonal IgG (1 : 50, Calbiochem/Millipore), anti-BrdU sheep polyclonal IgG (1 : 150, Abcam, Cambridge, England), anti-PDGFR-*α* rabbit monoclonal IgG (1 : 100, Santa Cruz Biotechnology, Santa Cruz, CA, USA), anti-MBP mouse monoclonal IgG (1 : 100, Chemicon), anti-Nestin mouse monoclonal IgM (1 : 200, Chemicon), anti-Olig2 rabbit polyclonal IgG (1 : 200, Chemicon), anti-GFP chicken polyclonal IgY (1 : 500, AVES, Aves Lab, Tigard, OR, USA), anti-GFP chicken polyclonal IgY (1 : 500, Calbiochem), anti-CD45 rat monoclonal IgG (1 : 500, BD Biosciences, San Diego, CA, USA). As secondary antibodies the following were used: rabbit anti-chicken biotin (1 : 200, Abcam), rabbit anti-chicken biotin (1 : 200, Vector Laboratories), goat anti-mouse biotin (1 : 200, Vector Laboratories), goat anti-mouse Alexa 488 (1 : 500, Molecular Probes), goat anti-mouse Alexa 594 (1 : 500, Molecular Probes), donkey anti-rabbit Alexa 488 (1 : 500, Molecular Probes), donkey anti-rabbit Alexa 594 (1 : 500, Molecular Probes), goat anti-rat biotin (1 : 200, Vector Laboratories), goat anti-rat Alexa 488 (1 : 500, Molecular Probes). 6-Diamidino-2-phenylindole (DAPI; 2 ng/ml; Molecular Probes) was used to stain nuclei. For non-fluorescent-conjugated antibodies, biotinylated secondary antibodies were visualized using the streptavidin–biotin–peroxidase method (Vectastain ABC Kit, Vector Laboratories), stained with the cromogen 3-3′-diaminobenzidine tetrahydrochloride (DAB Substrate Kit for peroxidase, Vector Laboratories) and counterstained with cresyl violet (Acros Organics, Geel, Belgium). Controls of the immunostaining were performed on two slides of each sample following the same procedure, but eliminating primary or secondary antibodies. Several microtome series were used to study the areas of demyelination in the cuprizone-treated mice. The sections were incubated with Luxol Fast Blue (1% in 95% ethanol with 0.5% acetic acid) and counterstained with Cresyl Violet according to the standard Klüver–Barrera protocol.^74^

### Microscopy and quantification

To obtain more precise and quantitative information, the 3D structure of the CC was analyzed separately. Inmunostained brain sections were divided into rostral (coronal sections with visible midline-crossing CC fibers) and caudal (coronal sections with visible non-midline-crossing CC fibers) for oligodendrocyte quantification analysis. Brain sections were observed under a fluorescence microscope (Leica DM6000D; Leica Microsystems). Micrographs were taken with DFC350/FX and DC500 Leica cameras. To quantify the number of Olig2+, NG2+, CC1+ and PDGFR-*α*+ cells, six to nine 14-*μ*m sections that were 168-*μ*m apart in the rostral CC, and six in the caudal CC, were randomly selected. The average cell number per section was calculated by counting labeled cells in five random 40X fields per section in the rostral CC and three random 40X fields per section in each hemisphere of the caudal CC (Figure 2j).

### MRI and myelin density quantification

The method used was similar to our previously published work.^66, 68^ Briefly, mice were injected with MSCs pre-cultured with 7 *μ*g/ml of Feraspin XL (Viscover, Miltenyi Biotec, Cologne, Germany). This allowed their detection using MRI. Mice were analyzed at different time points (0–15–30–60–90 days). To this end, mice were anesthesized with isoflurane (Esteve Veterinary), placing them in a fixed position in the apparatus. The mice were monitored throughout the whole procedure. Experiments were carried out in a horizontal 7 Tesla scanner with a 30 cm diameter bore (Biospec 70/30v; Bruker Medical, Ettlingen, Germany). The system had a 675 mT/m actively shielded gradient coil (Bruker Medical; BGA 12-S) of 11.4 cm inner diameter. A ^1^H mouse brain receive-only phase array coil with integrated combiner and preamplifier, no tune/no match, in combination with the actively detuned transmit-only resonator (Bruker BioSpin MRI) was used. Data were acquired with a Hewlett-Packard console running Paravision software (Bruker Medical) operating on a Linux platform.

In order to detect the cells, T2* multigradient echo images were acquired in the three orthogonal orientations with the following parameters: repetition time: 1.500 ms, echo time: 3 ms, flip angle: 30º, field of view: 20 × 20 mm, 20 slices, slice thickness: 0.5 mm, matrix: 256 × 256, two averages and a total acquisition time of 12 min 48 s.

To quantify the degree of remyelination of the CC over time, a ratio of mean T2 signal intensity of white matter to a reference region (cerebral cortex) was implemented. Myelin content within the CC was quantified by measuring the mean gray level with ImageJ software, obtaining the T2 ratio from every section, using a similar method as previously described.^75^ The average value was calculated for the rostral and caudal CC in both sham-operated and MSC-treated mice.

### Electrophysiological recording procedures

Axonal conduction velocity measurements were performed in a similar manner as previously described.^76^ Briefly, animals were killed by cervical dislocation at 2 or 4 months after transplantation or the equivalent age for sham treated and controls, brains were rapidly extracted and placed in a flask containing artificial CSF containing 124 mM NaCl, 2.5 mM KCl, 1.25 mM NaH_2_PO_4_, 2.5 mM MgCl_2_, 0.5 mM CaCl_2_, 26 mM NaHCO_3_ and 10 mM glucose (pH=7.4, osmolarity ≤300 mOsm/kg). Four 400-mm thick coronal slices, corresponding approximately to plates 25–31 and 40–46 in the atlas published by Franklin and Paxinos,^27^ were obtained and placed in a submersion type recording chamber, filled with extracellular solution at 37ºC, containing 124 mM NaCl, 5 mM KCl, 1.25 mM NaH_2_PO_4_, 1 mM MgCl_2_, 1.2 mM CaCl_2_, 26 mM NaHCO_3_ and 10 mM glucose (pH=7.4, osmolarity ≤300 mOsm/kg).

The CC was stimulated with a concentric bipolar electrode (Frederick Haer & Co., Bowdoin, ME, USA) using 0.1 ms pulses. Two simultaneous extracellular recordings were obtained with borosilicate glass microelectrode (0.2–5MΩ) filled with extracellular solution; the two electrodes were placed at a certain distance from each other as well as from the stimulating electrode (Figure 5a). The recordings were obtained at room temperature (24.3–26.3ºC). To enhance the signal-to-noise ratio, all the recordings were conducted on waveforms that were the average of 15 successive sweeps. For the recording analysis, Clampfit10.1 software (Axon Instruments, Molecular Devices, Sunnydale, CA, USA) was used. The conduction velocity was measured from the distance between the tips of both electrodes and the difference in the latency of the propagated action potential for both fast- and slow-conducting axons (Figure 5b).

### EM analysis

Mice were perfused with a 2% PFA, 2.5% glutaraldehyde fixative solution. The brains were dissected out and postfixed in the same solution. Coronal 200 *μ*m sections were cut on a vibratome (Leica VT-1000) and were postfixed with 2% osmium, rinsed, dehydrated and embedded in Durcupan resin (Sigma). Semithin sections (1.5 *μ*m) were cut, using an ultramicrotome (Leica EM UC-6) with a diamond-tipped knife (Histo; Diatome, Hatfield, PA, USA), and lightly stained with 1% toluidine blue to select the regions of interest. Ultra-thin sections (0.08 *μ*m) were obtained from the regions of interest, using an ultramicrotome (Leica EM UC-6) with a diamond-tipped knife (Ultra 45º Diatome), stained with Reynold's lead citrate (Reynolds Solution). The sections were observed under a transmission electron microscope (FEI Tecnai G2 Spirit BioTwin, Hillsboro, OR, USA) and images taken with a digital camera (Morada, Soft Imaging System, Olympus, Tokyo, Japan).

Three myelinated axon-rich telencephalic areas were studied: cingulate fascicle (cg), in the anterior levels of the telencephalon (from Bregma 1.10 to −0.10 mm); and the dorsal hippocampal commissure (dhc) and alveus of the hippocampus (alv), which are two regions of the posterior levels. These regions were chosen as their axons cut transversely in the same plane, are much more homogeneous than the CC, and therefore a more realistic G-ratio data can be obtained. Three levels for each region and animal were analyzed, and 100 myelinic axons from non-overlapping electron micrographs were measured per level in order to obtain the G-ratio analysis. The G-ratios of the axons were quantified using ImageJ software, implemented with a plug-in (http://gratio.efil.de/) to allow the semiautomated analysis of randomly selected sets of axons.^77^

### Statistical analysis

Statistical analysis of mean values was performed using Sigmaplot v12.0 (Systat Software, San Jose, CA, USA) and GraphPad Prism (San Diego, CA, USA) and SPSS softwares (IBM, Armonk, NY, USA). Statistical significance between control and experimental groups were calculated using Student's *t*-test for independent samples, one-way ANOVA and Bonferroni's multiple comparison *post hoc* test were performed where applicable, establishing the level of significance at *P*<0.05. Values are expressed as mean±S.E.M.

## Abbreviations

BBB: blood–brain barrier
BrdU: 5-bromo-2-deoxyuridine
CAP: compound axon potential
CC: corpus callosum
CM: conditioned medium
CSF: cerebrospinal fluid
EAE: experimental autoimmune encephalomyelitis
EM: electron microscopy
ERK: extracellular signal-regulated kinase
GFP: green florescent protein
IF: immunofluorescence
IHC: immunohistochemistry
LV: lateral ventricle
MAPK: mitogen-activated protein kinase
MBP: myelin basic protein
MRI: magnetic resonance imaging
MS: multiple sclerosis
MSC: mesenchymal stem cell
MSC-TX: mesenchymal stem cell transplanted
NSPC: neural stem progenitor cell
OPC: oligodendrocyte progenitor cell
PI3K: phospatidylinositol-3 kinase
SVZ: subventricular zone
V-SVZ: ventricular subventricular zone
