# Supplementary figures and images for: Intraventricular injections of mesenchymal stem cells activate endogenous functional remyelination in a chronic demyelinating murine model
Source: Cell Death Dis. 2016 May 12;7(5):e2223–. doi: 10.1038/cddis.2016.130 (PMC4917663; doi:10.1038/cddis.2016.130)

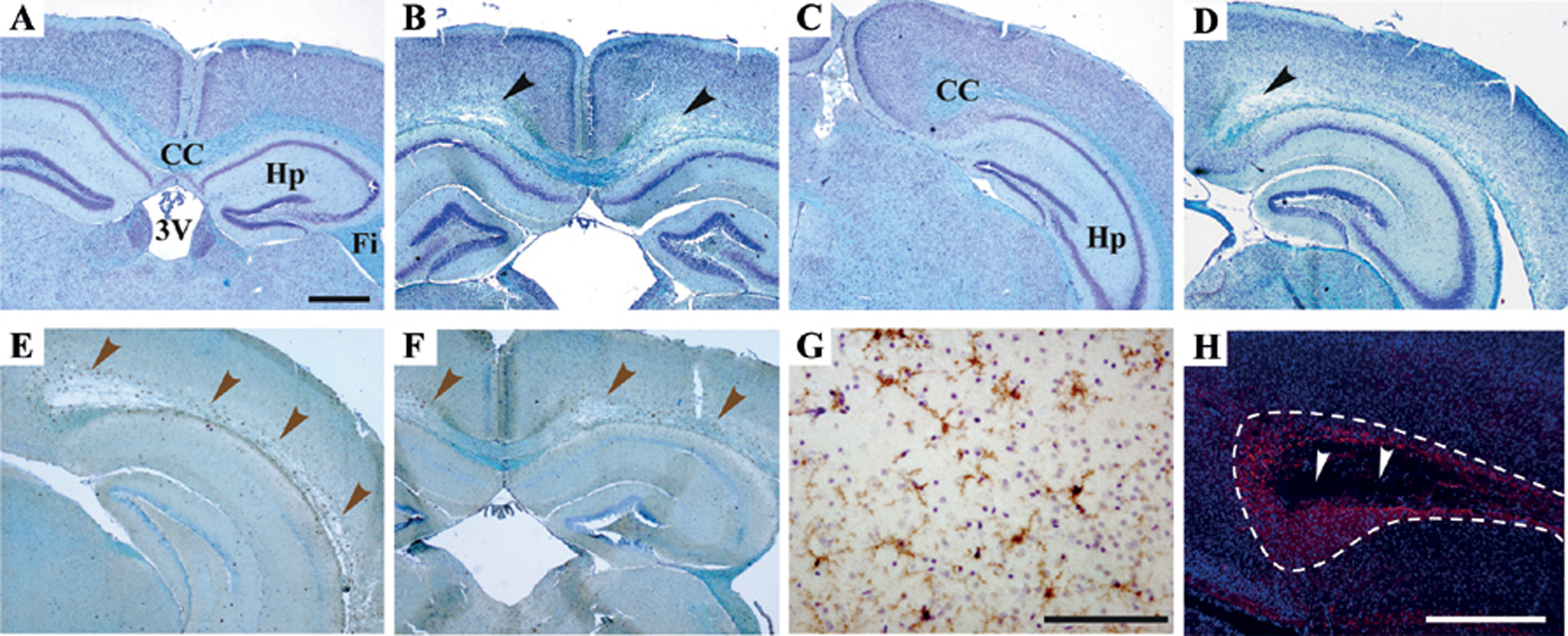

Supplement: Supplementary Figure S1 [file cddis2016130x1.tif]
