# Supplementary material for: Intraventricular injections of mesenchymal stem cells activate endogenous functional remyelination in a chronic demyelinating murine model
Source: Cell Death Dis. 2016 May 12;7(5):e2223–. doi: 10.1038/cddis.2016.130 (PMC4917663; doi:10.1038/cddis.2016.130)
Supplement: Supplementary Information [file cddis2016130x2.docx]

**Supplementary information.**

**Supplementary Table 1. List of primers used for the qPCR.**

**Supplementary Figure S1. Chronic cuprizone treatment caused severe demyelination in the CC.** Six-week old C57BL/6J mice were given cuprizone (CPZ, 0.2% w/w) in their diet for 12 weeks and then were euthanized and tissue was stained with Luxol Fast Blue (LFB) and counterstained with cresyl violet (Klüver-Barrera stainning) in all sections. Brain coronal sections from WT mice (A, C) and cuprizone-fed mice (B, D) were stained with Klüver-Barrera and the most rostral (midline-crossing) (A, B) and caudal CC (non midline-crossing) (C, D) sections were analysed. Black arrowheads indicate patches of demyelination (LFB-negative), similar to that observed in chronic lesions of multiple sclerosis. (E, F) Coronal brain sections of the CC were immunostained against NG2 (OPC marker) and stained with Klüver-Barrera. Brown arrowheads show patches of demyelination surrounded by high concentration of OPCs. (G) Higher magnification image representing the OPC NG2+ stellate morphology. (H) MBP staining (in red) of the CC (borders marked with a dotted line) of cuprizone-fed mice, counterstained with DAPI for nuclear staining (in blue). Abbreviations: CC, corpus callosum; Fi, fimbria; HP, hipocampus; 3V, third ventricle. Scale bar, 100 μm (A-F, H); 50μm (G).
